# Certain Autoimmune Manifestations Are Associated With Distinctive Karyotypes and Outcomes in Patients With Myelodysplastic Syndrome: A Retrospective Cohort Study

**DOI:** 10.1097/MD.0000000000003091

**Published:** 2016-04-01

**Authors:** Sang Jin Lee, Jin Kyun Park, Eun Young Lee, Sang Hyun Joo, Kyeong Cheon Jung, Eun Bong Lee, Yeong Wook Song, Sung-Soo Yoon

**Affiliations:** From the Division of Rheumatology (SJL, JKP, EYL, EBL, YWS), Seoul National University Hospital; Department of Molecular Medicine and Biopharmaceutical Sciences (SJL, JKP, YWS), Graduate School of Convergence Science and Technology, and College of Medicine, Medical Research Institute, Seoul National University, Seoul; Division of Rheumatology (SHJ), Chung Buk University Hospital, Cheongju, Department of Pathology (KCJ); and Division of Hematology and Oncology (S-SY), Seoul National University Hospital, Seoul, Korea.

## Abstract

Autoimmune manifestations (AIMs) are common in patients with myelodysplastic syndrome (MDS). This study aimed to investigate whether AIMs are associated with a specific cytogenetic abnormalities and worse survival in patients with MDS.

A total of 67 MDS patients with AIMs and 134 age- and sex-matched MDS patients without AIMs, all of whom received medical care at Seoul National University Hospital from January 2000 through July 2014, were enrolled. The clinical features, chromosomal abnormalities, and outcomes were examined. The effect of AIMs on mortality was estimated after adjusting for age, sex, and the International Prognostic Scoring System.

The mean age (±SD) at the time of MDS diagnosis was 54.5 ± 17.1 years, and 44.8% of patients were male. Neutrophilic dermatosis (ND; Sweet syndrome and pyoderma gangrenosum) was the most prevalent AIM (n = 24 36%]), followed by Behcet disease (10 [15%]), rheumatoid arthritis (9 [13%]), vasculitis (8 [12%]), myositis (3 [4%]), spondyloarthropathy (3 [4%]), and systemic lupus erythematous (2 [3%]). ND and vasculitis occurred at the time of MDS diagnosis, whereas other AIMs occurred years after MDS diagnosis. Deletion of 5q was associated with ND (*P* = 0.001), whereas trisomy 8 was associated with Behcet disease (*P* = 0.015). Strikingly, ND was associated with a 1.8-fold increase in mortality (95% CI 1.033–3.093; *P* = 0.038).

Certain AIMs in MDS patients are associated with distinctive karyotypes and worse survival. A larger study is needed to confirm whether the presence of AIMs influences disease outcome in MDS.

## INTRODUCTION

Myelodysplastic syndrome (MDS) is a disease entity encompassing a heterogeneous group of hematopoietic disorders characterized by impaired generation and maturation of hematopoietic cells in the bone marrow, with subsequent peripheral blood cytopenia. The natural history of MDS is diverse, ranging from asymptomatic indolent disease to death owing to bone marrow failure or progression to leukemia.^1^

In general, 10% to 20% of patients with MDS develop autoimmune manifestations (AIMs), which include neutrophilic dermatosis (ND) (eg Sweet syndrome and pyoderma gangrenosum), systemic lupus erythematosus (SLE), rheumatoid arthritis (RA), relapsing polychondritis, Behcet disease (BD), and vasculitis involving large-, medium-, and small-sized vessels.^2–6^ Interestingly, BD associated with MDS is often associated with severe intestinal ulcers and trisomy 8.^7,8^

At present, it is unclear whether AIMs are associated with a worse outcome for MDS patients. Enright et al reported that MDS patients with AIMs had a significantly worse prognosis than those without AIMs,^2,3^ whereas others report that AIMs do not affect the prognosis.^4–6^ A recent report by Farah et al identified the presence of skin lesions as a risk factor for transformation of MDS into acute myeloid leukemia (AML).^9^

Since cytogenetic instability owing to accumulating mutations is a key step during MDS pathogenesis, cytogenetic abnormalities are a key prognostic factor in the International Prognostic Scoring System (IPSS), which also takes into account the percentage of bone marrow blasts and the number of cytopenias.^10^ The presence of abnormalities on chromosome 7 or a complex karyotype (ie, ≥3 abnormalities) are associated with a worse prognosis, whereas a normal karyotype, an isolated 5q deletion, an isolated 20q deletion, or an isolated-Y suggest a better prognosis.^10^ To date, little is known about the association between AIMs and cytogenetic abnormalities.^11^ Here, we examined whether AIMs are associated with a specific karyotype and prognosis in MDS patients.

## METHODS

### Patients

A total of 67 MDS patients with AIMs who received medical care at Seoul National University Hospital from January 2000 through July 2014 were enrolled in this retrospective cohort study. MDS was diagnosed according to the WHO classification.^12^ Exclusion criteria were patients who were younger than 18 years, who were initially diagnosed with aplastic anemia or leukemia, and who developed MDS after a chemotherapy (supplemental Figure 1). As a reference group, 134 age- and sex-matched MDS patients without AIMs, who were treated during the same period in the same hospital, were randomly selected from the electronic medical record archive (ie, AIMs to non-AIMs ratio, 1:2). Baseline clinical characteristics, bone marrow pathology, karyotype, and IPSS were ascertained by chart review. Follow-up began at the time of MDS diagnosis and was censored at the time of death or at the date of the last follow-up, whichever came first. Survival status was confirmed using National Death Register from the Korea Statistics Promotion Institute (www.stat.or.kr).

The study was approved by the Institutional Review Board (IRB) at Seoul National University Hospital. The requirement for informed consent was waived by the IRB as the study involved a minimum risk to the enrolled patients and no identifiable information was used.

### Chromosomal Analysis

For cytogenetic analysis, metaphases were evaluated according to the International System for Human Cytogenetic Nomenclature (2013).^13^ Chromosomal aberrations were determined by fluorescence in situ hybridization (Abbott Laboratories, Chicago, IL).

### Histology of AIMs

Nineteen biopsy samples taken from AIM sites in the enrolled patients were available from the pathology archive and reviewed. An independent pathologist (KCJ) evaluated the cytohistological features of the infiltrating inflammatory cells.

### Statistical Analysis

Continuous variables were expressed as the mean ± SD and categorical variables as percentages. The former were assessed using Student *t* test or the Mann-Whitney test, and the latter were assessed using the *χ*^2^ test or Fisher exact test, as appropriate. Survival curves were generated using the Kaplan-Meier method and compared using the log rank test. Multivariable Cox proportion hazard ratio (HR) models were used to estimate the relative risk of death. *P* values ≤0.05 were considered statistically significant. All statistical analyses were performed using IBM SPSS (statistics version 19.0, Chicago, IL).

## RESULTS

### Clinical Characteristics of the MDS Patients

At the time of diagnosis, the mean age of MDS patients with AIMs was 54.5 ± 17.1 years, and 44.8% were male. The mean follow-up duration after MDS diagnosis was 40.7 ± 45.5 months (Table 1). There was no difference between MDS patients with AIMs and the reference group with respect to MDS subtype, IPSS, or MDS treatment.

**TABLE 1:**
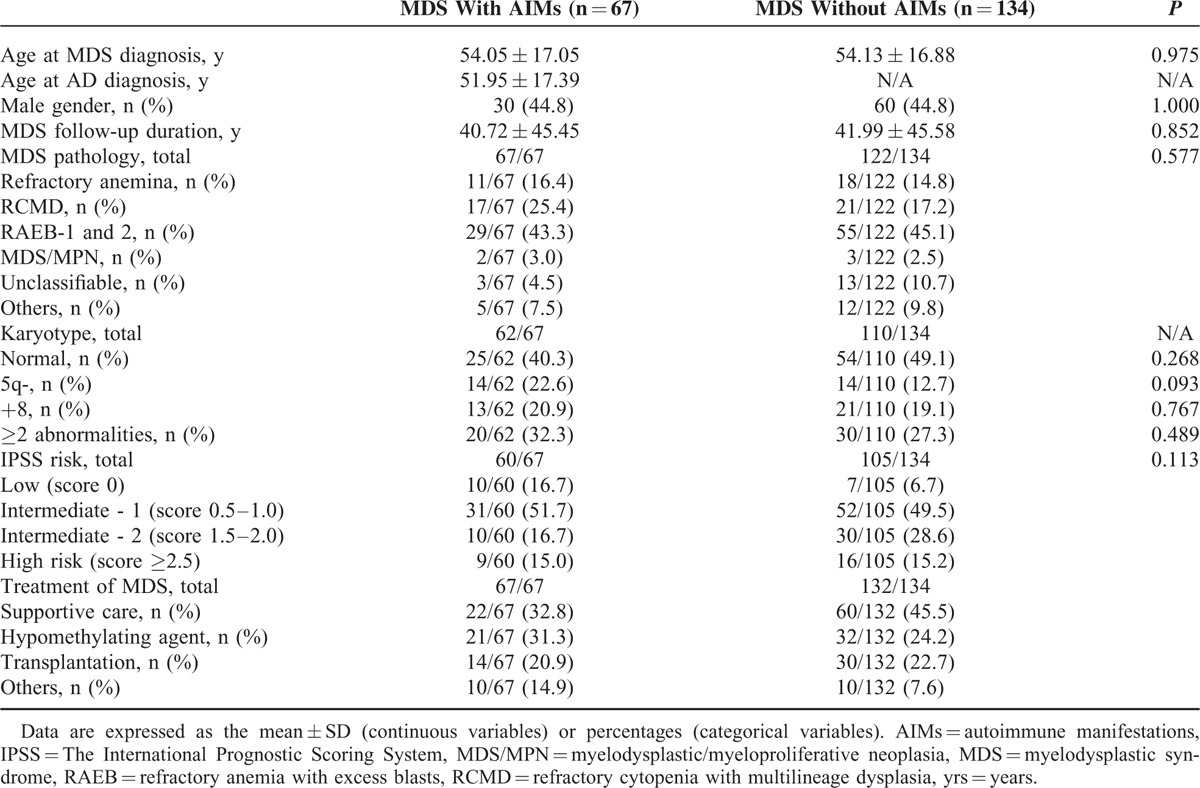
Baseline Characteristics of MDS Patients With and Without AIMs

### AIMs Associated With MDS

**FIGURE 1:**
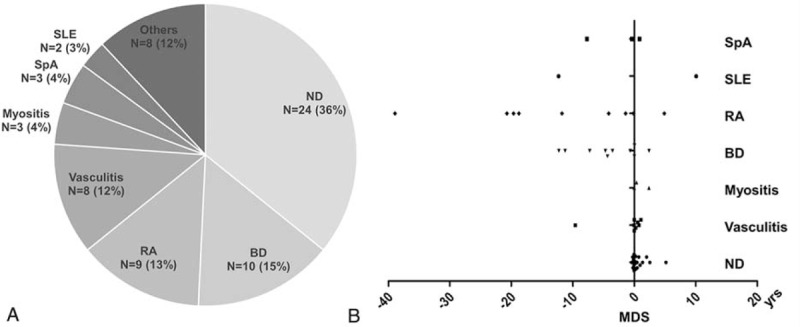
Distribution of AIMs in 67 MDS patients (A) and time from AIM diagnosis to MDS (B). AIM = autoimmune manifestations, BD = Behcet disease, MDS = myelodysplastic syndrome, ND = neutrophilic dermatosis, RA = rheumatoid arthritis, SLE = systemic lupus erythematous, SpA = spondyloarthropathy, yrs = years.

ND (including Sweet syndrome [n = 22] and pyoderma gangrenosum [n = 2]) was the most common AIM (24 [35.8%] of 67 observed cases), followed by BD (10 [14.9%]), RA (9 [13.4%]), vasculitis (including 2 cases involving large vessels, 1 involving medium-sized vessels, and 5 cases of cutaneous vasculitis) (8 [11.9%]), myositis (3 [4.5%]), spondyloarthropathy (SpA) (3 [4.5%]), and SLE [2 [3.0%]) (Figure 1A). Interestingly, 7 (70.0%) of the 10 BD patients developed severe colitis. Panniculitis (n = 2), Sjogren syndrome (n = 2), systemic sclerosis (n = 1), chronic demyelination encephalopathy (n = 1), migratory arthritis (n = 1), and relapsing polychondritis (n = 1) were rare. Of note, 10 of the 67 (14.9%) patients had ≥2 AIMs.

The time between diagnosis of MDS and that of AIMs varied markedly. Myositis, vasculitis, and ND developed concomitantly with (or shortly after) MDS diagnosis, whereas BD and RA developed years before MDS (Figure 1B). SpA and SLE did not appear to bear any discernible temporal relationship to MDS.

### Association Between AIMs and Karyotype

**FIGURE 2:**
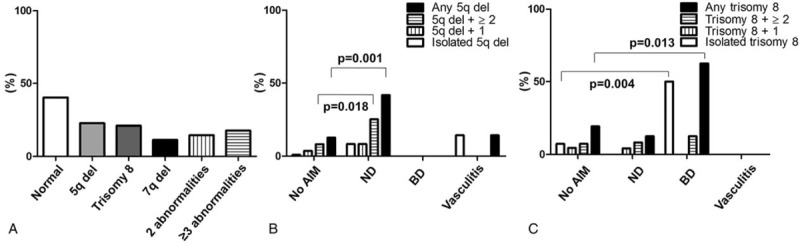
Comparison of karyotypes in MDS patients with and without AIMs. (A) Distribution of chromosomal abnormalities in MDS patients with AIMs. (B) Comparison between MDS patients with and without AIMs in terms of 5q deletion (B) or trisomy 8 (C), either alone or in conjunction with additional karyotypic abnormalities. AIMs = autoimmune manifestations, BD = Behcet disease, Del = deletion, ND = neutrophilic dermatosis.

Karyotype data were available for 62 (92.5%) of the 67 MDS patients with AIMs and for 110 (82.1%) of the 134 matched MDS patients without AIMs. The karyotype was normal in 25 (40.3%) of the 62 MDS patients with AIMs and in 54 (49.1%) of the 110 MDS patients without AIMs. The most common chromosomal abnormality in MDS patients with AIMs was 5q deletion (n = 14, 22.6%), followed by trisomy 8 (n = 13, 20.9%) (Figure 2A).

Significantly more MDS patients with ND had a 5q deletion than those without AIMs (41.7% vs 12.7%, *P* = 0.001). Interestingly, for the 80% of MDS patients with ND and a 5q deletion, the deletion was present in combination with ≥1 additional chromosomal abnormalities. By contrast, the rate of 5q deletion in MDS patients with BD was similar to that in the reference group without AIMs (Figure 2B).

Trisomy 8 was present in 5 (62.5%) of the 8 MDS with BD compared with 21 (19.1%) of the 110 MDS without AIMs (*P* = 0.013). Only 1 (20%) of the 5 MDS patients with BD had another chromosomal abnormality in addition to trisomy 8. The prevalence of trisomy 8 was not higher in MDS patients with ND or vasculitis than in those without AIMs (Figure 2C).

### Cancer Cells Are Rare at AIM Sites

The striking temporal relationship between MDS and some AIMs raises a question of whether some autoimmune features are caused by direct leukemic infiltrates rather than by an autoimmune inflammatory response. Tissue infiltration by malignant cells was evaluated at 19 AIM sites. Only one (5.3%) of the 19 available biopsies showed evidence of infiltration by large blastic leukemic cells (Supplementary Figure).

### Association Between AIMs and Outcome for MDS Patients

**FIGURE 3:**
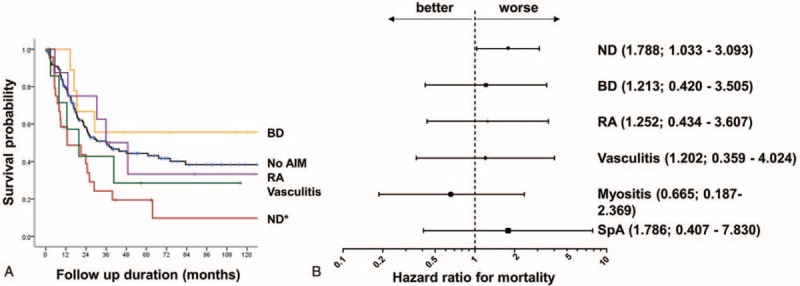
Survival of MDS patients with different AIMs. (A) Kaplan-Meier survival curves. ∗*P* = 0.003 denotes the difference in survival between MDS patients with neutrophilic dermatosis (ND) and MDS patients with no AIMs. (B) Cox hazard ratio for mortality after adjusting for age, sex, and IPSS. Hazard ratios and 95% confidence intervals are shown in brackets (B). BD = Behcet disease, ND = neutrophilic dermatosis, RA = rheumatoid arthritis, SpA = spondyloarthropathy, SLE = systemic lupus erythematous, IPSS = The International Prognostic Scoring System.

There was no difference between MDS patients with and without AIMs in terms of overall survival (*P* = 0.308, log rank test). Subgroup analysis according to AIM during the follow-up period revealed that the death rate among MDS patients with ND was higher than that among MDS patients without AIMs (*P* = 0.003). The survival rates of MDS patients with BD, vasculitis, or RA were no different from those of MDS patients without AIMs (Figure 3A).

After adjusting for age, sex, and IPSS, ND remained an independent factor associated with a significantly higher risk of death (HR, 1.788; 95% CI, 1.033–3.093; *P* = 0.038) (Figure 3B).

## DISCUSSION

Autoimmunity arises because of an imbalance between the mechanisms that stimulate and inhibit innate and adaptive immune responses. The fine balance between activating and inhibiting signals that regulate immune cells is influenced by signaling cascades at the cellular level. Thus, certain allelic polymorphisms or de novo mutations in the genes that regulate inflammatory responses constitute a risk factor for developing not only cancer but also rheumatic diseases.^14,15^ Conceivably, the generation of dysfunctional innate cells due to cytogenetic instability might initiate and perpetuate inflammatory cascades, leading to the development of AIMs in MDS patients, particularly when the counterbalancing anti-inflammatory responses are compromised.^11^

The clinical spectrum of chronic immunologic diseases is determined by the relative contributions of dysfunctional innate and adaptive immune cells to inflammation in affected organs.^16^ Impaired adaptive immune cells promote the “autoimmune spectrum,” which includes RA and SLE, whereas a disturbance in innate cells causes the “autoinflammatory” spectrum of immunological diseases, which includes ND and inflammatory bowel disease.^17,18^ The striking temporal relationship between ND and MDS diagnosis observed in the present study supports the notion that MDS (and the associated production of dysfunctional innate cells) contributes to the higher incidence of ND as an “autoinflammatory AIM.” It is possible that MDS itself may increase the risk of classic autoimmune diseases such as RA and SLE, as MDS patients have high numbers of proinflammatory T helper 17 cells and regulatory T cells.^19,20^ However, the absence of a temporal association with SLE or RA suggests that their coexistence might be just coincidental. Inflammatory myositis showed a tight temporal association with MDS; this was expected since myositis of a paraneoplastic nature commonly manifests within 1 to 2 years of cancer diagnosis.^21^

Interestingly, although most BD cases occurred years before MDS diagnosis, 5 (62.5%) of the 8 MDS patients with BD had trisomy 8. It is tempting to speculate that this subset of BD patients with a cytogenetic abnormality reflected an early paraneoplastic AIM that preceded MDS diagnosis by years. Indeed, gain-of-function of the *PTPN11* gene with a subsequent increase in the reactivity of innate immune cells has been reported in a BD patient with MDS.^22^ It might be of interest to investigate whether “subkaryotypic” cytogenetic abnormalities or genetic polymorphisms are present in different clinical subsets of BD disease in the absence of MDS.

A key pathological process in MDS is mutation of genetic material; therefore, cytogenetic abnormalities are an important prognostic indicator.^10^ Approximately 60% of patients with AIMs had an abnormal karyotype. In the present study, chromosome 5q deletion was most common abnormality and was present in 22.6% of MDS patients with AIMs. Consistent with previous observations, 5q deletion exists in combination with other chromosomal abnormalities in 85% of cases.^23,24^ As ND was tightly associated with 5q deletion, the (haplo) insufficiency of proteins encoded by chromosome 5q might be responsible for an augmented immune response. Indeed, 5q deletion is associated with sustained NF-kappa B signaling,^25^ which activates expression of genes involved in proinflammatory responses and apoptosis.^26,27^

The tight temporal relationship between MDS and certain AIMs raises the question of whether aggressive and dysfunctional malignant myeloid cells infiltrate extramedullary organs and clinically resemble an AIM. However, malignant cells were found only in 1 (5.3%) of 19 examined tissues (Supplemental Figure 2), confirming that AIMs in MDS are an autoimmune rather than a leukemic phenomenon.

Data on the effect of AIMs on MDS survival are scarce and conflicting. Previous studies report no significant differences in survival between MDS patients with and without AIMs^5,6^ although a small study of 15 patients found that the presence of cutaneous AIMs tended to be associated with a higher risk of leukemic transformation.^9^ Here, we showed that the survival of MDS patients with ND was significantly worse than that for MDS patients without AIMs. The poorer survival of MDS patients with ND might be related to the presence of the complex karyotype associated with 5q deletion, which increases mortality.^28,29^ However, after adjusting for age, sex, and IPSS risk, ND itself remained associated with an approximately 1.8-fold increased risk of death when compared with the reference group, strongly suggesting that ND is an independent risk factor for a poor outcome. Therefore, the emergence of ND may mark a critical level of cytogenetic instability that drives MDS progression, with a subsequently poor prognosis.

This retrospective study has several limitations. First, the cohort drawn from a single center is relatively small, meaning that the confidence intervals are wide. Second, the present study design does not take into account the effects of MDS treatment on cancer outcome, as cancer treatments vary widely. Third, the mechanism(s) by which AIMs influence MDS outcome requires further investigation. A larger prospective study is needed to confirm our findings.

In conclusion, certain AIMs are associated with a particular cytogenetic abnormality in patients with MDS. In addition, ND was an independent predictor for a worse outcome for MDS patients. Further studies are needed to clarify the mechanism(s) driving MDS and AIMs.

## Supplementary Material

Supplemental Digital Content
