# Supplementary material for: Certain Autoimmune Manifestations Are Associated With Distinctive Karyotypes and Outcomes in Patients With Myelodysplastic Syndrome: A Retrospective Cohort Study
Source: Medicine (Baltimore). 2016 Apr 1;95(13):e3091. doi: 10.1097/MD.0000000000003091 (PMC4998533; doi:10.1097/MD.0000000000003091)
Supplement: Supplemental Digital Content [file medi-95-e3091-s001.doc]

**Supplemental figure 1.** **Flow chart for exclusion and inclusion criteria**


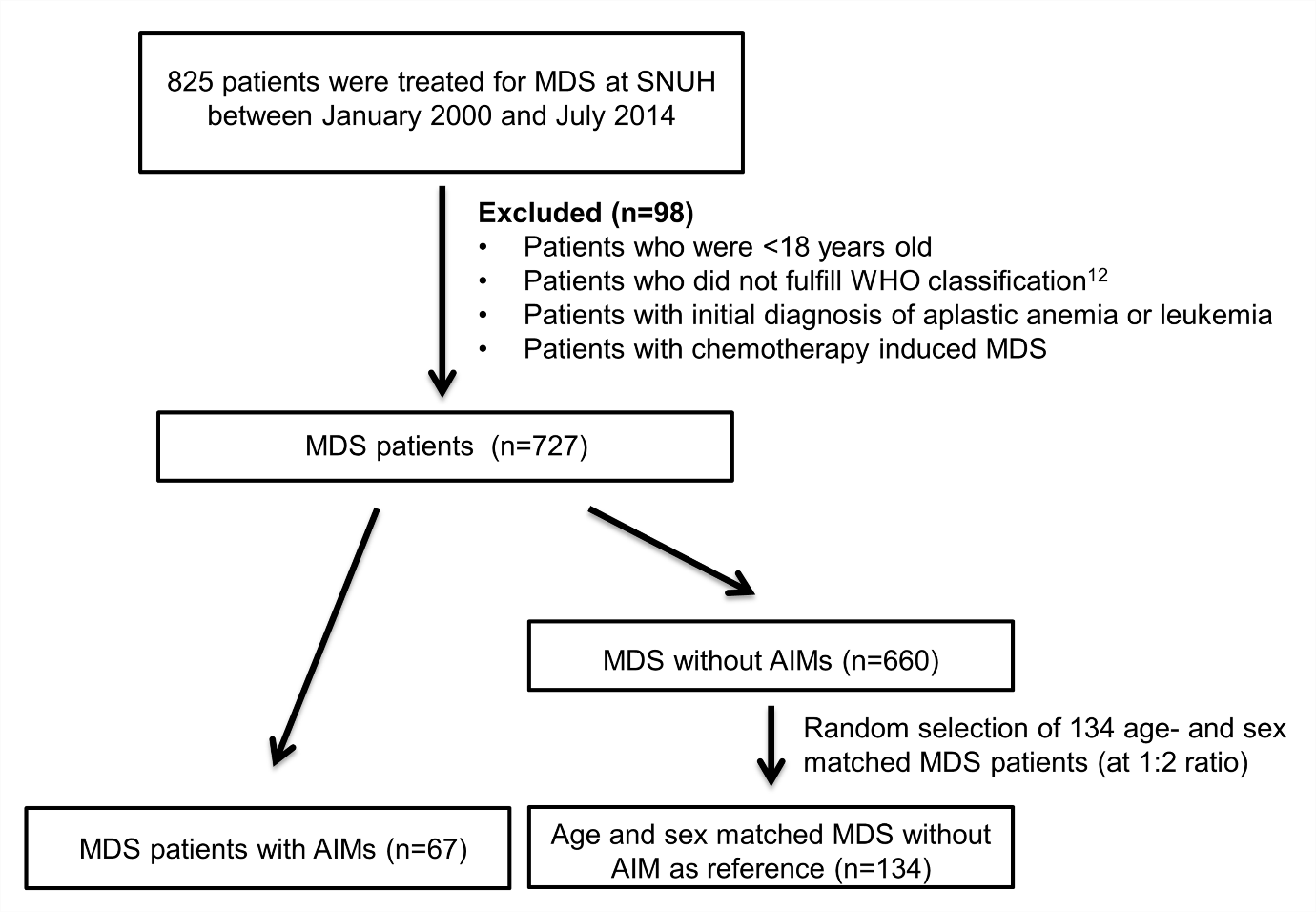


**Supplemental figure 2.** **Neutrophilic dermatosis and MDS involvement in the skin of an MDS patient.** Skin sections from an MDS patient with an AIM (magnification, ×400). A representative example of neutrophilic dermatosis (A) and MDS involvement (B) in the skin. Arrows show blast cells stained positive for myeloperoxidase. MDS = myelodysplastic syndrome; AIM= autoimmune manifestation; ND = neutrophilic dermatosis.


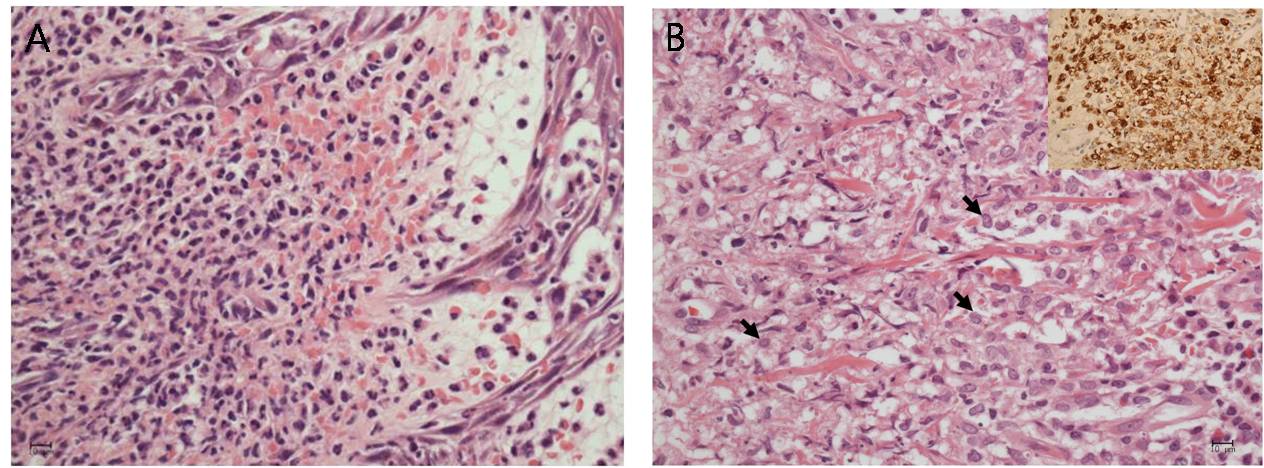


Supplemental table 1. Biopsy of autoimmune manifestation sites (AIMs).

| **Case** | **AIM** | **Biopsy**  **site** | **Infiltrating cells** |
| --- | --- | --- | --- |
| **#1** | ND | Skin | Lymphocytic and neutrophilic infiltration |
| **#2** | ND | Skin | Lymphohistiocytic and neutrophilic infiltration |
| **#3** | ND | Skin | Lymphocytic and neutrophilic infiltration |
| **#4** | ND | Skin | Diffuse neutrophilic infiltration |
| **#5** | ND | Skin | Diffuse neutrophilic infiltration |
| **#6** | ND | Skin | Diffuse neutrophilic infiltration |
| **#7** | ND | Skin | Lymphocytic and neutrophilic infiltration |
| **#8** | ND | Skin | Perivascular lymphocytic infiltration |
| **#9** | ND | Skin | Mild perivascular lymphohistiocytic infiltration |
| **#10** | ND | Skin | Perivascular and perieccrine neutrophilic infiltration |
| **#11** | ND | Skin | Superficial perivascular lymphocytic infiltration |
| **#12** | ND | Skin | Mild perivascular lymphocytic infiltration |
| **#13** | ND | Skin | Furuncle with lymphocytic and neutrophilic infiltration |
| **#14** | ND | Skin | MDS involvement with large blastic leukemic cells |
| **#15** | BD colitis | Ileum | Chronic active inflammation with lymphocytic, neutrophilic and eosinophilic infiltration |
| **#16** | BD colitis | Skin | Lymphocytic and neutrophilic infiltration |
| **#17** | BD colitis | Colon | Chronic active inflammation with lymphocytic and neutrophilic infiltration |
| **#18** | Myositis | Muscle | Segmental myonecrosis with mild lymphocytic and eosinophilic infiltration |
| **#19** | Myositis | Muscle | Abscess with heavy neutrophilic infiltration |

AIM = autoimmune manifestations; BD = Behcet’s disease; ND = neutrophilic dermatosis; Dx = diagnosis.
